# Trends in socioeconomic inequalities in smoking in Turkey from 2008 to 2016

**DOI:** 10.1186/s12889-021-12200-x

**Published:** 2021-11-20

**Authors:** Hur Hassoy, Isil Ergin, Gorkem Yararbas

**Affiliations:** 1 Department of Public Health, Ege University Faculty of Medicine, Izmir, Turkey; 2 Institute on Drug Abuse, Toxicology and Pharmaceutical Science, Ege University, Izmir, Turkey

**Keywords:** Smoking, Socioeconomic inequalities, Smoking epidemic model, Global adult tobacco survey

## Abstract

**Background:**

Smoking inequalities in Turkey were previously demonstrated in an early stage of the smoking epidemic model. This paper aimed to assess the trends for socioeconomic inequalities in smoking in Turkey over the years in the context of the smoking epidemic model using data from the Global Adult Tobacco Survey (GATS) Turkey 2008–2012-2016.

**Methods:**

Cross-sectional data were analyzed to calculate the association of smoking with, wealth, education, occupation and place of residence using age-standardized prevalence rates, odds ratios, relative index of inequality (RII) and slope index of inequality (SII). The analysis was performed separately for age groups (younger: 20–39 years/older: 40 and above years) and sex.

**Results:**

Younger women with higher wealth and older women with higher wealth and education smoked more. For both age groups, smoking was increased for working class and urban women. Relative wealth inequalities in smoking narrowed and then showed a reversal for younger women (RII_2008_ = 3.37; 95% CI:1.64–3.40; RII_2012_ = 2.19; 95% CI:1.48–3.24; RII_2016_ = 0.80; 95% CI:0.58–1.10, p-for trend < 0.0001). Relative educational inequalities in smoking for older women also showed a narrowing (RII_2008_ = 21.45; 95% CI:11.74–39.19; RII_2012_ = 15.25; 95% CI:9.10–25.55; and RII_2016_ = 5.48; 95% CI:3.86–7.78, p-for trend < 0.0001). For older women, a similar narrowing was observed for wealth (RII_2008_ = 3.94; 95% CI:2.38–6.53; RII_2012_ = 2.79; 95% CI:1.80–4.32; and RII_2016_ = 1.34; 95% CI:0.94–1.91, p-for trend = 0.0001). The only significant trend for absolute inequalities was for younger women by wealth. This trend showed a narrowing and then a reversal (SII_2008_ = 0.14; 95% CI:0.09–1.20; SII_2012_ = 0.12; 95% CI:0.06–0.18; and SII_2016_ = -0.05; 95% CI:-0.12–0.02, p-for trend = 0.0001). Unlike women, smoking in men showed inverse associations for wealth and education, although not statistically confirmed for all years. Smoking was increased in working classes and unemployed men in 2012 and 2016. Inequalities did not show a trend in relative and absolute terms for men.

**Conclusions:**

For smoking inequalities in Turkey, a transition to the next stage was observed, although the previously defined Southern European pattern also existed. Low socioeconomic women deserve special attention as well as stressors at work and drivers of smoking at urban settings.

**Supplementary Information:**

The online version contains supplementary material available at 10.1186/s12889-021-12200-x.

## Background

Smoking is one of the major factors contributing to health inequalities. Lopez [1] defined the diffusion of smoking in countries based on the four-stage “smoking epidemic model”. In the first stage, the smoking prevalence is low in both sexes. In the following stage, the prevalence increases steadily among men. The smoking prevalence peak in the third stage is followed by a decrease. Women follow this pattern with a delay of approximately 20 years. In the last stage, the smoking prevalence declines and reaches a stable point for both sexes. Subsequent work linked this model to socioeconomic inequalities in smoking. In the earlier stages, smoking is more common in higher socioeconomic groups for both sexes. In the latter stages, smoking becomes more common in lower socioeconomic groups, whereas the overall smoking prevalence declines [2, 3].

Socioeconomic inequalities in smoking differ by sex and country. Education, income and wealth contribute both independently and together to socioeconomic inequalities in smoking [4–6]. The patterns of high smoking risk in women in low socioeconomic groups in Northern European countries and in high socioeconomic groups in Southern European countries have been referred to as reverse inequalities or positive gradients [2, 4, 7, 8]. Patterns among younger (20–39 years old) and older (40 years old and above) generations may also vary. Younger generations are the first to abandon smoking behavior, initially among men and then among women [4, 9].

Socioeconomic inequalities in smoking have rarely been studied in Turkey. The study analyzing World Health Survey 2002 data showed an almost even distribution of socioeconomic variances among men across all regions. Additionally, the study found that women with higher income and educational attainment smoked more. Patterns of socioeconomic inequalities in smoking behavior in Turkey were similar to those observed in Southern European countries. The inequalities associated with smoking in Turkey were consistent with the patterns estimated by the smoking epidemic model, and Turkey was in the early stages compared to Northern European countries. Characteristics such as modernity, emancipation, and independence, which may be particularly associated with smoking among women, were highlighted [10].

M-POWER is a policy package developed by the World Health Organization (WHO) aimed at helping to implement effective country-level interventions to reduce tobacco demand. Since 2010, important steps have also been taken to implement M-POWER policies [11] in Turkey. A series of anti-smoking measures, such as making all indoor areas (restaurants, coffee houses and bars) smoke-free and introducing smoking cessation hotlines and polyclinics, were implemented for tobacco control in Turkey. The effects of such implementation need to be evaluated with regard to socioeconomic inequalities. It would be interesting to determine how the socioeconomic inequalities associated with smoking have been transformed in the aftermath of the immediate implementation of these nationwide tobacco-control practices. Identifying this association during the period covered by the Global Adult Tobacco Survey (GATS) data may be a revelation for other countries going through the same stages.

This paper aimed to assess the trends for socioeconomic inequalities in smoking in Turkey over the years in the context of the smoking epidemic model. We looked for answers to the following questions: does the Southern European pattern continue, or do we see signs of progress towards the next stage in the smoking epidemic in terms of socioeconomic inequalities? We tested these research questions using the GATS 2008, 2012 and 2016 datasets for Turkey.

## Methods

### Data

The GATS is a global standard protocol for systematically monitoring adult tobacco use and tracking key tobacco control indicators [12]. In Turkey, the GATS was first conducted in 2008 and repeated in 2012 and 2016. The GATS is a household survey of persons who are 15 years of age and older, and it uses multistage stratified cluster sample designs to produce nationally representative data. We analyzed the GATS 2008, 2012 and 2016 country data for Turkey, with the permission of the World Health Organization’s Country Office in Turkey. For the GATS 2008, 2012 and 2016 surveys, 11,200, 11,536 and 11,200 households were sampled, respectively. One individual was randomly selected from each participating household to complete the survey. The overall response rate of the completed individual interviews for the 2008, 2012 and 2016 surveys were 9030 (90.9%), 9851 (90.1%), and 8760 (82.2%), respectively [13–15]. Respondents below 20 years of age, occasional smokers and those with missing data for the socioeconomic variables were excluded from the analysis. Details about the data excluded from the study for each year are presented in Supplementary Table 1. The remaining data for the GATS 2008, 2012 and 2016 surveys belonging to 8178, 8915 and 8115 individuals respectively were used for analyses.

### Variables

Age, sex, place of residence, wealth, educational attainment, and occupation were included in the analysis as independent variables. Age was measured in years. To enable the assessment of different patterns for younger and older generations shown in previous studies [2, 3, 5, 8, 10], the study group was divided into two age groups: a “younger” age group (20–39 years) and an “older” age group (≥40 years). This segmentation was conducted to compare generations that show different trends in terms of socioeconomic inequalities. Place of residence were grouped as “urban” and “rural”, as in the GATS surveys. Wealth was defined using ownership data on electricity, flush toilet, sewage connection, fixed telephone, cell telephone, television, radio, refrigerator, washing machine, car, and a moped/scooter/motorcycle [13–15]. The answers to these 11 items were used to calculate a household wealth score ranging from 0 to 11. Those who had an item scored 1 point, while those who did not possess the item scored 0 points. The resulting total scores were classified into five groups: 10–11: “highest”; 9: “second highest”; 8: “middle”; 7: “second lowest”; and 0–6: “lowest”. For the present analysis, we used a classification that resulted in a reasonable number of respondents in each wealth group. Educational attainment was determined based on educational achievements. Those who identified themselves as “illiterate”, “only literate” and “not graduated” were classified into the low educational attainment group. In Turkey, primary school, middle school and high school each take 4 years. “Elementary school”, “primary education” and “secondary or vocational secondary school graduates” were classified into the moderate educational attainment group. “High school or equivalent”, “college or faculty” and “master’s/doctorate degree” holders were classified into the higher educational attainment group. Occupational position was determined by the main employment status over the past 12 months. The occupation groups were unemployed, nongovernment employee, self-employed, government employee, homemaker, retired, and student. For the purposes of the analysis, the homemaker, retired and student groups were combined.

Current daily smoking served as the dependent variable, in accordance with the literature [5–7]. “Occasional smokers” are an unstable and heterogeneous group [16] and differ from current daily smokers in terms of socioeconomic status and health consequences related to smoking [5]. Regarding these limitations, we excluded these groups as they were a small group in our databases. This exclusion was also performed in the study by Schaap and Kunst [17]. In the GATS questionnaire, current daily smokers were identified by responses to the following question: “Do you currently smoke tobacco on a daily basis, less than on a daily basis, or not at all?” Respondents who confirmed smoking tobacco on a “daily basis” were classified as current daily smokers. The smoking variable was categorized as current daily smoker and non-smoker (never smokers + former smokers).

### Statistical analysis

Age-standardized prevalence rates and their confidence intervals were calculated using the direct method of standardization, with a distinction per 5-year age group. The European standard population 2013 was used as the standard population [18]. Crude and age-standardized prevalences were provided in Supplementary Fig. 1.

The trends in socioeconomic inequalities in smoking over the years were measured using odds ratios (ORs), the relative index of inequality  (RII), and the slope index of inequality (SII). The association of smoking with wealth, educational attainment and occupation, logistic regression analysis was applied. The lowest educational attainment, the lowest wealth group and the homemaker/retired/student group served as the reference categories. In these analyses, the associations for wealth, educational attainment, occupation and place of residence were measured separately for age group (20–39 years/40 and above years) and sex. For the comparisons between the younger and older age groups, we used regression models that controlled only for age. The results are presented as odds ratios with 95% confidence intervals in Supplementary Table 2 and 3.

The prevalence rates were used for the calculation of the RII and SII to obtain a summary measure of both relative and absolute inequalities and to take into account the changing cumulative distribution of the population for each socioeconomic indicator. The population in each education and wealth category is assigned a ridit score based on the midpoint of the range in the cumulative distribution of the population in the category. The analysis for each socioeconomic indicator was performed using the highest and lowest socioeconomic groups by gender and age groups, respectively. The RII can be interpreted as a relative risk (the ratio of the prevalence of the risk factor among the persons with the lowest socioeconomic group compared with those with the highest) whereas the SII can be interpreted as the risk difference between the two. However, smoking presents reverse inequalities especially for women among Southern European countries [8]. Thus, to ease interpretation, the reference group has been assigned as low socioeconomic group. Log-binomial regression model was used with logarithmic and identity link functions for calculations of RIIs and SII, respectively. RII/SII models were fitted in SAS (9.4 TS Level 1 M6) and calculations were performed by sex  and age group for education and wealth indicators, separately. *P* values for trend were presented for significancy [19, 20]. IBM SPSS Statistics for Windows, version 25.0 (IBM Corp., Armonk, N.Y., USA) was used for logistic regression analysis.

## Results

Table 1 shows the distribution of the surveyed population according to socioeconomic indicators, age groups and sex and for the GATS 2008, 2012 and 2016 Turkey data. With regard to wealth groups for 2008, 2012 and 2016, the majority of the surveyed population was in the middle wealth group (25.3, 27.8 and 32.1%, respectively). While the middle and second highest wealth groups expanded over the years, the lowest wealth group in 2016 decreased to 2.9%. Wealth data were predominantly similar in both genders and age groups. With regard to educational attainment, the study population was predominantly at the moderate educational attainment level for all years and both sexes. Over the years, it was observed that while the number of people with higher education has increased for both genders, the number with low education has decreased. Young people were in the higher education group and the elderly were in the low education group for all years and both sexes. With regard to occupation, the majority of women (73.2–89.7%) were predominantly in the homemaker/retired/student group for all years and age groups, whereas young men and men in total were predominantly in the nongovernmental employee group (33.1–59.6%). With regard to place of residence, rural-urban distribution was approximately equal in both sexes and in the total for 2008 and 2012. In 2016, the population living in urban areas increased to approximately 90% in both sexes, age groups and in total.

**Table 1:** The distribution of the surveyed population (in column % of the total population) according to socioeconomic indicators, sex and age groups, GATS 2008, 2012 and 2016 Turkey

	2008			2012			2016		
	Women Y/O/T (%) ***N*** = 3854	Men Y/O/T (%) ***N*** = 4324	Total Y/O/T (%) ***N*** = 8178	Women Y/O/T (%) ***N*** = 4951	Men Y/O/T (%) ***N*** = 3964	Total Y/O/T (%) ***N*** = 8915	Women Y/O/T (%) ***N*** = 4118	Men Y/O/T (%) ***N*** = 3997	Total Y/O/T (%) ***N*** = 8115
**Wealth Groups**									
Highest	16.1/14.6/15.3	17.0/19.1/18.2	16.5/16.8/16.7	12.0/12.4/12.2	12.8/15.9/14.6	12.3/14.0/13.3	13.3/13.7/13.5	14.1/14.8/14.4	13.7/14.2/14.0
Second highest	25.2/25.1/25.2	25.4/25.3/25.4	25.3/23.8/24.3	24.7/22.6/23.4	23.9/27.2/25.9	24.3/24.6/24.5	30.1/31.6/30.9	32.2/31.2/31.7	31.2/31.4/31.3
Middle	24.4/24.1/24.3	25.7/23.5/24.4	25.0/25.8/25.3	29.6/26.7/27.8	29.4/26.7/27.8	29.5/26.7/27.8	31.3/33.3/32.4	30.6/33.4/31.9	30.9/33.3/32.1
Second lowest	19.1/16.5/17.7	18.9/15.8/17.1	19.0/16.2/17.4	25.5/21.8/23.3	24.9/18.4/21.0	25.2/20.3/22.2	22.8/17.8/20.0	20.3/18.2/19.3	21.4/17.9/19.7
Lowest	15.1/19.6/17.6	13.0/16.3/14.9	14.2/18.0/16.3	8.3/16.5/13.2	9.1/11.9/10.8	8.6/14.5/12.1	2.5/3.6/3.1	2.8/2.5/2.7	2.7/3.1/2.9
**Education Groups**									
High education	26.5/8.6/16.8	41.9/18.6/28.3	33.4/13.5/22.2	37.5/10.5/21.3	51.2/22.8/34.2	43.6/16.0/27.0	54.8/18.9/35.1	68.7/29.2/50.6	62.3/23.5/42.7
Moderate education	61.1/44.6/52.2	55.1/67.9/62.6	58.4/56.1/57.1	52.1/47.1/49.1	46.4/65.3/57.7	49.6/55.2/52.9	42.3/63.9/54.1	31.0/66.6/47.3	36.2/65.1/50.8
Low education	12.4/46.7/31.0	3.0/13.5/9.1	8.2/30.4/20.7	10.3/42.4/29.6	2.3/12.0/8.1	6.8/28.9/20.0	2.9/17.2/10.7	0.3/4.2/2.1	1.5/11.4/6.5
**Occupation Groups**									
Unemployed	1.1/6.7/4.1	8.5/36.0/24.6	4.4/21.1/13.8	1.9/0.3/1.0	8.0/2.6/4.8	4.6/1.3/2.6	2.5/0.5/1.4	7.9/4.9/6.5	5.4/2.5/3.9
Nongovernment employee	14.8/4.8/9.4	59.6/23.5/38.4	34.6/14.0/23.0	14.9/3.5/8.1	52.3/20.2/33.1	31.6/10.9/19.2	14.6/6.2/10.0	53.6/29.5/42.5	35.6/16.6/26.0
Self-employed	2.4/2.7/2.6	26.4/35.8/31.9	13.0/18.9/16.3	4.0/4.6/4.4	18.3/22.3/20.7	10.4/12.5/11.6	1.6/1.6/1.6	12.9/13.9/13.3	7.7/7.1/7.4
Government employee	–	–	–	6.0/1.8/3.5	14.0/9.3/11.2	9.6/5.1/6.9	6.0/2.0/3.8	10.4/7.7/9.2	8.3/4.6/6.5
Homemaker/retired/student	81.7/85.7/83.9	5.5/4.6/5.0	48.0/46.0/46.9	73.2/89.7/83.1	7.4/45.6/30.3	43.8/70.2/59.6	75.4/89.7/83.2	15.2/44.0/28.4	43.0/69.2/56.2
**Place of Residence**									
Urban	43.5/55.2/49.8	41.4/55.9/49.9	42.5/55.6/49.9	59.5/42.8/49.4	58.8/43.7/49.7	59.2/43.2/49.6	92.0/87.6/89.6	91.4/85.7/88.8	91.7/86.8/89.2
Rural	56.5/44.8/50.2	58.6/44.1/50.1	57.5/44.4/50.1	40.5/57.2/50.6	41.2/56.3/50.3	40.8/55.6/50.4	8.0/12.4/10.4	8.6/14.3/11.2	8.3/13.2/10.8

*Y* Younger (20–39 years), *O* Older (40 years and above), *T* Total.

**Fig. 1:**
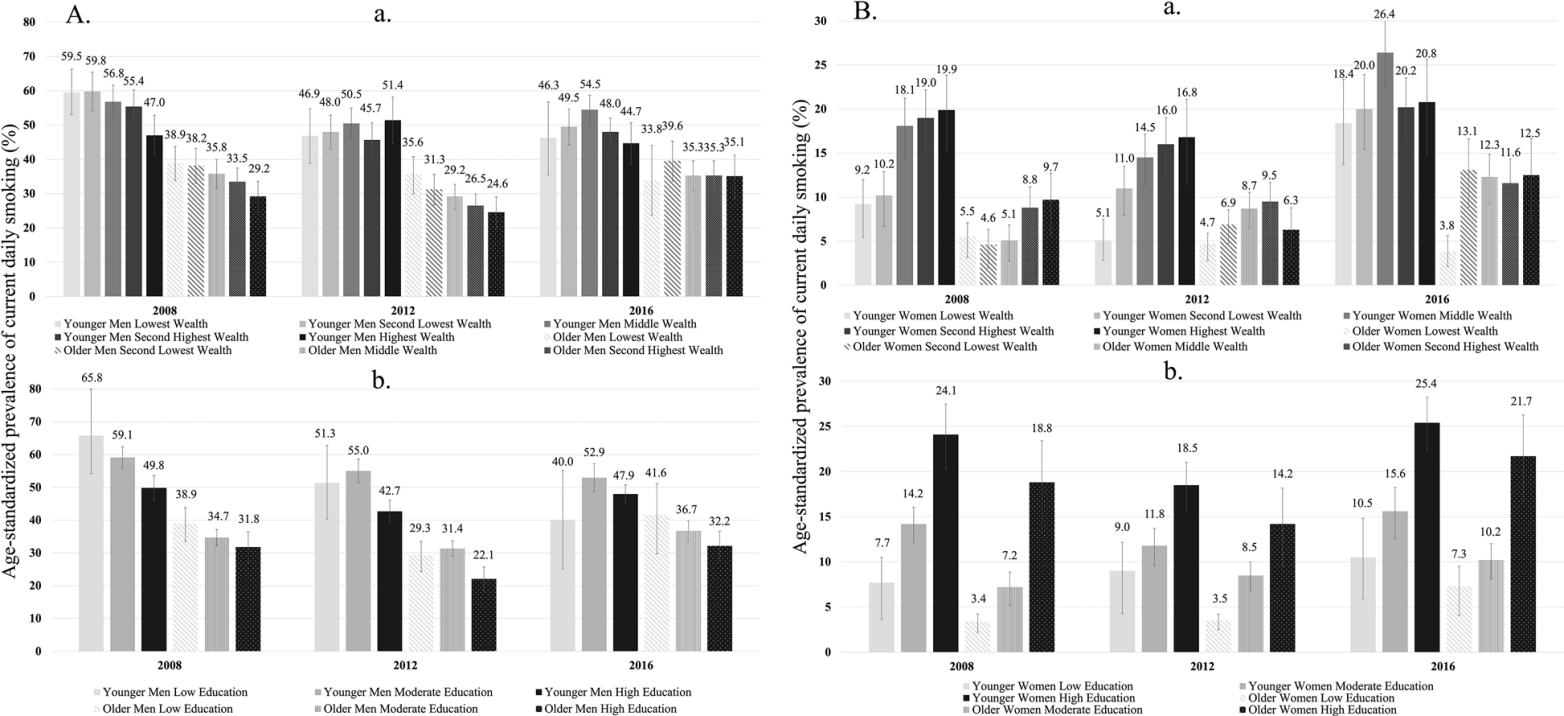
**A** Age-standardized prevalence with 95% confidence intervals of current daily smoking status among men by wealth (a) and education (b) per year and age group (%). **B** Age-standardized prevalence with 95% confidence intervals of current daily smoking status among women by wealth (a) and education (b) per year and age group (%)

Age-standardized prevalence with 95% confidence intervals of current daily smoking status among men by wealth (a) and education (b) per year and age group are presented in Fig. 1A. (The national crude and age standardized prevalences for each age group per sex between 2008 and 2016 are in Supplementary Fig. 1).

For wealth; among the younger men, the decreasing gradient for smoking prevalence from the lowest to highest wealth groups (59.5, 59.8, 56.8, 55.4 and 47.0%) observed in 2008 was not repeated in 2012 and 2016. The same pattern was also observed among the older men (38.9, 38.2, 35.8, 33.5 and 29.2%) in 2008 and (35.6, 31.3, 29.2, 26.5 and 24.6%) in 2012, which disappeared in 2016.

For education; among the younger men, current daily smoking prevalence decreased in the low (65.8, 51.3 and 40.0%) and moderate (59.1, 55.0 and 52.9%) educated groups (2008, 2012 and 2016 respectively), throughout the years. Among the older men, the decreasing gradient from the lowest to highest wealth groups (38.9, 34.7 and 31.8%) was observed in 2008 and (41.6, 36.7 and 32.2%) 2016 and this pattern was not repeated in 2012.

Age-standardized prevalence with 95% confidence intervals of current daily smoking status among women by wealth (a) and education (b) per year and age group are presented in Fig. 1B.

For wealth; the prevalence of smoking increased gradually from lower to the higher wealth groups in 2008 and 2012 (with an exception at the highest wealth group of older women in 2012). This gradient disappeared in 2016. At 2016, the prevalence of smoking has increased for all wealth groups (except for the lowest wealth group of older women).

For educational attainment; the prevalence of smoking was increasing from lower education group to the higher education. This was a consistent pattern for both age groups throughout the years. A clear gradient was observed in each year with low educated having the lowest prevalence. The lowest educated women showed a consistently increasing pattern with 7.7, 9.0 and 10.5% for younger women and 3.4, 3.5 and 7.3% for older women for current daily smoking between 2008 and 2016. The highest prevalences were at highly educated women in each year for both age groups: An inconsistent pattern was observed as 24.1, 18.5 and 25.4% among younger women, 18.8, 14.2 and 21.7% among older women for 2008, 2012 and 2016, respectively.

Supplementary Table 2 and 3 present the ORs for current daily smoking by wealth, education, occupation, and place of residence among men and women in the two age groups. For younger women by wealth, the ORs increased gradually from the lowest to the highest wealth groups in 2008 and in 2012 and no statistically significant difference was observed in 2016. For education, the ORs showed increasing gradients from the low to the high educated women for both age groups with a diminishing trend in the extent of inequalities throughout the years and being a nongovernmental employee significantly increased the odds of smoking in the younger and older women. For the older women; in 2016, the self-employed and governmental employees showed significant differences. Regarding the urban-rural comparison, urban dwellers had increased odds compared with their rural counterparts. Among men, for wealth and education, there were inverse associations in comparison with those detected for women, which was not statistically confirmed for all years. For occupation; unemployed, nongovernmental employee and self-employed men showed increased OR’s for smoking. Urban-rural comparison showed no statistically significant difference for smoking throughout the years.

**Fig. 2:**
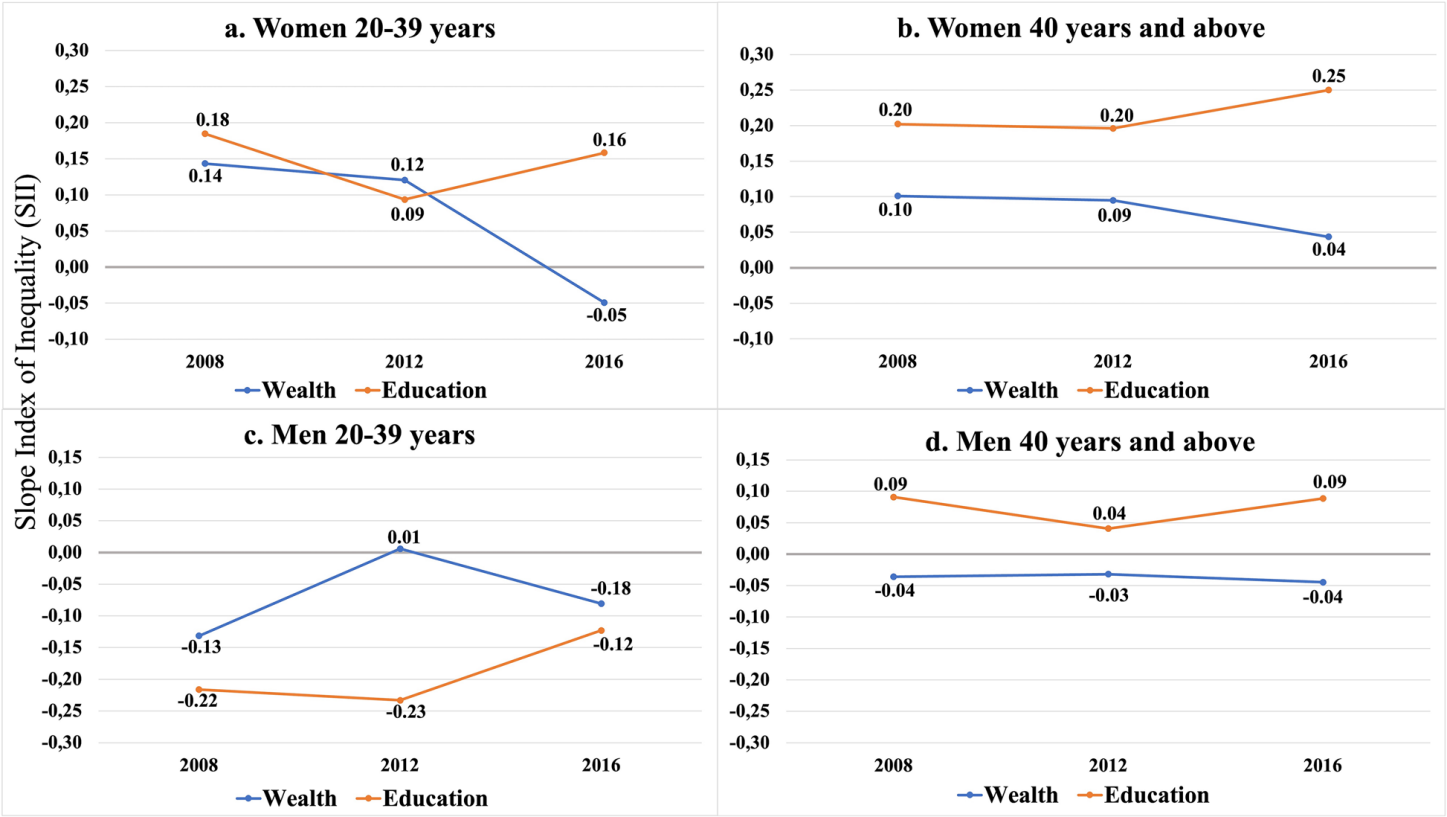
Relative index of inequalities (RII) of age adjusted current daily smoking prevalence by socioeconomic indicators, sex, age groups and survey years

The trends for RIIs of age-adjusted current daily smoking prevalences by socioeconomic indicators, sex and age groups are presented in Fig. 2. There were significant interactions for 20–39 year-old women by wealth (*P* < 0.001) (Fig. 2a) and 40+ women by education level (P < 0.001) and wealth (*P* = 0.001) (Fig. 2b). For younger women by wealth (Fig. 2a), a narrowing for smoking inequalities in relative terms for 2008 (RII = 3.37; 95% CI: 1.64–3.40) to 2012 (RII = 2.19; 95% CI: 1.48–3.24) was observed and this tended to reverse in 2016 (RII = 0.80; 95% CI: 0.58–1.10). For older women by education level (Fig. 2b); RIIs diminished significantly in 2008 (RII = 21.45; 95% CI: 11.74–39.19), 2012 (RII = 15.25; 95% CI: 9.10–25.55) and 2016 (RII = 5.48; 95% CI: 3.86–7.78). The same was observed for wealth and the RIIs were attenuated over time; (RII = 3.94; 95% CI: 2.38–6.53) in 2008, (RII = 2.79; 95% CI: 1.80–4.32) in 2012 and (RII = 1.34; 95% CI: 0.94–1.91) in 2016. For all groups, men showed no significant interaction for time (Fig. 2c and d).

**Fig. 3:**
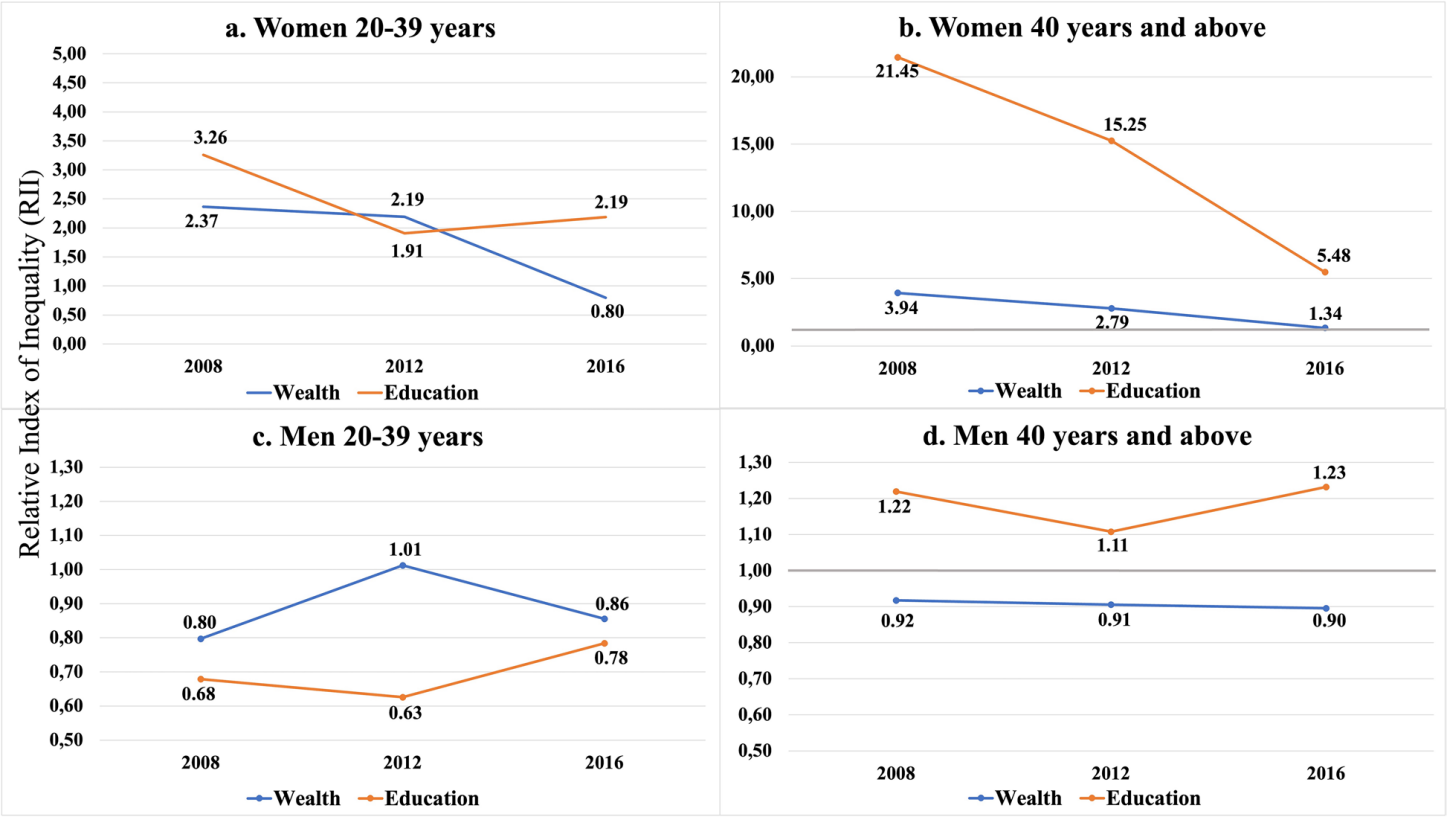
Slope of Inequality index (SII) of age adjusted current daily smoking prevalence by socioeconomic indicators, sex, age groups and survey years

The trends for SII of age-adjusted current daily smoking prevalences by socioeconomic indicators, sex and age groups are presented in Fig. 3. There was one significant interaction detected among the groups. It was for 20–39-year-old women by wealth (*P* < 0.001) (Fig. 3a). For younger women by wealth, a narrowing for absolute inequalities in smoking for 2008 (SII = 0.14; 95% CI: 0.09–1.20) to 2012 (SII = 0.12; 95% CI: 0.06–0.18) was observed and this tended to reverse in 2016 (SII = − 0.05; 95% CI: − 0.12–0.02). For all groups, men showed no significant interaction for time (Fig. 2c and d).

The results in Figs. 2 and 3 were parallel for all groups except for older women by education (Fig. 2 b and Fig. 3 b). The smoking inequality in relative terms (RII) was significantly narrowing while the smoking inequality in absolute terms (SII) was tending to increase although, insignificant. This could be attributed to the doubling prevalences throughout the years among the low educated group with low rates of smoking (Fig. 1). Because this group had a numerically low prevalence, the changes in inequalities in absolute terms did not significantly appear as the change in inequalities in relative terms.

## Discussion

This study analyzed the trends of the socioeconomic inequalities in smoking in Turkey in the context of the smoking epidemic model based on the GATS 2008–2012-2016 data. The changes in the patterns of inequalities over the years were investigated. We identified signs of progress towards the next stage in the smoking epidemic in relative and absolute terms of socioeconomic inequalities.

Educational attainment was the most important determinant of the risk of smoking among older women for all year sections although the extent of inequalities was narrowing. Among younger women, smoking prevalence was initially lower in the higher educational strata, but over time, the inequalities leveled out. In a similar pattern, wealth-related inequalities narrowed, and then reversed in both relative and absolute terms. These findings could be interpreted as signs of a transition to the next stage in the smoking epidemic in terms of socioeconomic inequalities. However, the risk of smoking is still elevated for both younger and older working-class women and for women who live in urban settings. These findings were parallel with the Southern European pattern. Unlike women, smoking in men showed inverse associations for wealth and education, although not statistically confirmed for all years. There were also significant differences concerning occupation. The highest risk was observed in the unemployed group and working-class men. Smoking inequalities have increased over the years for unemployed men. Wealth and educational inequalities did not show a trend in relative and absolute terms for men.

### Strengths and limitations

Nationally representative datasets with high response rates of three different time sections were used in the study herein. Moreover, it was the first study assessing the trends of smoking inequalities over time in Turkey.

Underreporting in the investigation of smoking may have occurred; however, most studies have found self-reports on smoking to be reliable, consistent and accurate [21, 22]. We may consider that the investigation of smoking behavior based on self-reports may be biased, especially among women, due to the influence of cultural patterns in countries such as Turkey. Even though this method of data collection may potentially create bias, we may assume that the associations identified for inequalities cannot be entirely linked to this issue. The lack of the “region” variable in GATS data is a limitation in revealing regional inequalities. The “paid employed” group for the occupation variable in 2008 data, was renamed into two groups, “governmental employee” and “nongovernmental employee”, in the 2012 and 2016 data. Although this change created limitations in terms of comparison, it also created an opportunity to examine private and public sector employees. Combining homemaker, retired and student groups may cause bias. If there were information on the household head’s occupation status (for the homemaker or the student) or the previous job (for the retired) a better categorization would be possible. However, the data lacks this detail. Regarding the similarity that, they do not occupy a role in the labor market, they have been merged. Considering the small number of students and retired individuals, however, this impact is likely negligible. The urban-rural population distribution in the 2008 and 2012 data changed substantially in the 2016 data. This was due to the change in legal municipal borders in the relevant years and should not be interpreted as a sampling problem.

### Interpretation of results and comparison with the literature

In the present study, high educational attainment stood out as the primary determinant of an increased risk of smoking, especially among older women for all years. This was parallel to the positive gradient that was previously documented among older women in Southern Europe and the Eastern Mediterranean [8]. Smoking may still resemble a symbol of liberation and freedom for educated older women [23]. On the other hand, the lower smoking risk among lower educated older women may be related to the social position of women in their families, the patriarchal nature of society, traditional stereotypes, and religion rather than reflecting a higher level of health awareness. These factors may protect women with low educational attainment from smoking [24]. However, for older women, the narrowing in RII and the small increase in SII can be explained by the doubling prevalences throughout the years among the low educated group with low rates of smoking. Because this group had a numerically low prevalence, the changes in inequalities in absolute terms did not significantly appear as the change in inequalities in relative terms. This signals an increasing smoking behavior among low educated women which has been defined previously as a consequence of factors such as inequitable access to smoking cessation services and marketing strategies of tobacco industry [3]. The higher acceptability of smoking among low socio-economic women may also be interpreted as the conservative environment losing its power against smoking [10] and the growing emancipation overcoming the resistance to female smoking [8]. Among highly educated younger women, the significant odds of smoking in the highest education group disappeared in the 2012 data, which may be considered an important implication for the transition to the next phase of the smoking epidemic when evaluated together with the diminishing relative inequality for older women throughout the years. The pattern in which educated women smoke more, is first abandoned by younger generations. In the meantime, among the older educated generation, inequality diminishes but persists. Parallel to the interpretation of the smoking epidemic model, it may be predicted that throughout the years, the burden of smoking will be gradually delivered to low education groups, adding substantially to health inequalities.

The gradual change in inequalities has also been examined for wealth. For women, it has been observed that wealth-related inequalities narrow for both older and younger groups, and they are reversed for younger women. The findings of the 2002 WHS data for Turkey describe a major role of wealth in smoking among women [10]. In the latter years covered in this study, the role of wealth changed for both age groups. This gradually disappearing role may again signify the transition to the next phase. Thus, the previously described Southern European pattern (positive gradient) (increased smoking prevalence among women who have higher levels of wealth) [8] no longer exists for younger women.

The odds of smoking were elevated among working women for all three- time sections for both age groups, particularly among nongovernmental and paid employees. Additionally, the scale of the inequality remained largely unchanged across the compared age groups. Studies have shown that stress is the primary factor that increases smoking prevalence [25] and that high levels of stress are associated with increased smoking prevalence, especially among women [26]. The work environment and fast pace of the private sector may constitute contributing factors to stress and consequently to an increased prevalence of smoking. Additionally, the work environment and high job demands may be associated with smoking [27]. It was found that the smoking prevalence among women increased along with the pace of their work schedules [28].

The study results showed that the smoking prevalence increased among urban women in comparison with their rural counterparts for both age groups. This is parallel to Völzke’s finding which revealed that metropolitan communities smoked more at all ages than rural communities. This relation between urbanicity and smoking was more pronounced among women than men. This increased risk was attributed to stressful life in urban areas: work-related conditions with long working hours, fear of unemployment, pressure for performance at large companies, diminished social relations and increased effort for social contacts [29]. Schulz et al. conceptualize this as “environmental demands” going beyond adaptive capacity. These conditions are probably more likely to trigger stress among women than men [30]. Under stress, people may engage in behaviors that are detrimental to their health [29]. The study results showed that even after adjustment for wealth and education, urbanicity is a determinant for smoking among women. However, the diminished risk in 2012 in the younger age group may be a signal of transferring this risk to rural counterparts. A Polish study observed such a reversed trend and attributed this shift to increased quitting in urban areas [31]. This may imply the abundance of interventions in urban areas and increased access to these interventions (cessation programs, banning advertisements or other restrictions), from which rural dwellers may not benefit. Thus, it should be emphasized that tobacco control interventions should not create new gaps across the social gradient.

Among men, unlike women, a decreasing gradient for smoking prevalence from the lowest to highest wealth and education groups was observed in some years. Higher tobacco consumption in low-income and low-education men has been detected previously. In line with the diffusion of the smoking epidemic, smoking becomes more associated with lower socioeconomic status. This has been explained by less concern for the future health risks with prioritization of immediate reward, earlier smoking initiation, barriers for access to cessation services and resources and lower success rates for quitting attempts among lower socioeconomic men. Conventional health education efforts and tobacco control policies neglecting the disadvantaged groups also add to these [2, 6]. However, these associations among men in the study herein, were not as strong and consistent as women signaling the effect of gender identity among men. This gender identity for smoking implies an important role of smoking that goes beyond socioeconomic issues and brings men of different backgrounds together. It seems to be an entrenched part of men’s identity. This is not surprising given the long- standing efforts of the tobacco industry to prescribe what was “masculine” and what was “feminine”. The relationship that was constructed long ago between respectable masculinities and smoking still seems to survive thrive [32, 33]. A biomedical approach that neglects this social and political context will fail to prevent and control smoking epidemics. Thus, the social roles, identities, and influence of gender, deserve special attention in tackling this epidemic and the health inequalities it creates [34]. For men, the role of occupation in smoking also differs for different groups. Unemployed, nongovernmental and self-employed men have an increased risk of smoking and have higher odds than others. Unemployment is not only a job loss but also the loss of many psychological functions, including identity and self-esteem [35]. With the economic and social losses that it triggers, unemployment may pave the way for depression and negative behavioral changes and increase the number of cigarettes smoked [36]. A countrywide representative study in Iraq found that unemployment was the most significant risk factor for current daily smoking [37]. When assessing the increased risk among these working classes in 2012 and 2016, the economic crisis of 2008 should be considered. Thus, in the post-economic crisis period, the group of nongovernmental employees may suffer from low income, and self-employed individuals may be threatened by income insecurity [38]. These groups have previously been associated with work stress, competition in the workplace and fear of job loss [39]. Al Badri’s study showed that nongovernmental employees and the self-employed had the highest risk of smoking [37]. The economic crisis may have contributed to the already high stressors among these groups. This indicates the importance of the previously conceptualized broader understanding of health determinants and health inequalities encompassing socioeconomic, cultural, and environmental conditions [40]. The occupational status related to these stressors seems to be an important contributor to inequalities in smoking. Smoking may be a coping strategy as a stress reliever or a means of reinforcement of masculine identity, that has been damaged by job loss, job insecurity or low pay.

## Conclusions

Wealth- and education- related inequalities narrowed among older women and even reversed for wealth among the younger women. These changes in the patterns of inequalities, could be interpreted as evidence of the transition to the next phase in the smoking epidemic model regarding inequalities. However, the odds of smoking were persistently elevated throughout the years for working-class women and for women living in urban settings. These findings were still parallel with the previously defined Southern European pattern. In addition, unemployed and working-class men were found to be at a higher risk of smoking.

To address the smoking epidemic, specific attention needs to be paid to examining the stressors experienced in the work environment and urbanity. Moreover, socioeconomic instabilities such as economic crises which exacerbate these stressors, need to be evaluated with regard to their psychological impact. Unemployment also deserves special attention in this context. For the smoking codes entrenched in men’s identity, a sociopolitical context is necessary to attack the ongoing efforts of the tobacco industry.

With these findings and in light of the smoking epidemic model, it may be predicted that throughout the years, the burden of smoking will be gradually delivered to low socioeconomic groups especially for women. Health inequalities that already exist for these groups will be exacerbated by the increased smoking risk.

## Supplementary Information

**Additional file 1: Supplementary Figure 1** Crude and age-standardized prevalence with 95%CIs of current daily smoking status by sex, age group and year.

**Additional file 2: Supplementary Table 1** The details about the data excluded from the study for each year.

**Additional file 3: Supplementary Table 2** Odds ratios for current daily smoking for women according to socioeconomic indicators by age group and survey year.

**Additional file 4: Supplementary Table 3** Odds ratios for current daily smoking for men according to socioeconomic indicators by age group and survey year.

## Data Availability

The datasets used and/or analyzed during the current study are available from the corresponding author on request.

## Abbreviations

GATS: Global Adult Tobacco Survey
WHO: World Health Organization
OR: Odds ratio
RII: Relative index of inequality
SII: Slope index of inequality
