# Supplementary figures and images for: Trends in socioeconomic inequalities in smoking in Turkey from 2008 to 2016
Source: BMC Public Health. 2021 Nov 20;21:2128. doi: 10.1186/s12889-021-12200-x (PMC8605534; doi:10.1186/s12889-021-12200-x)

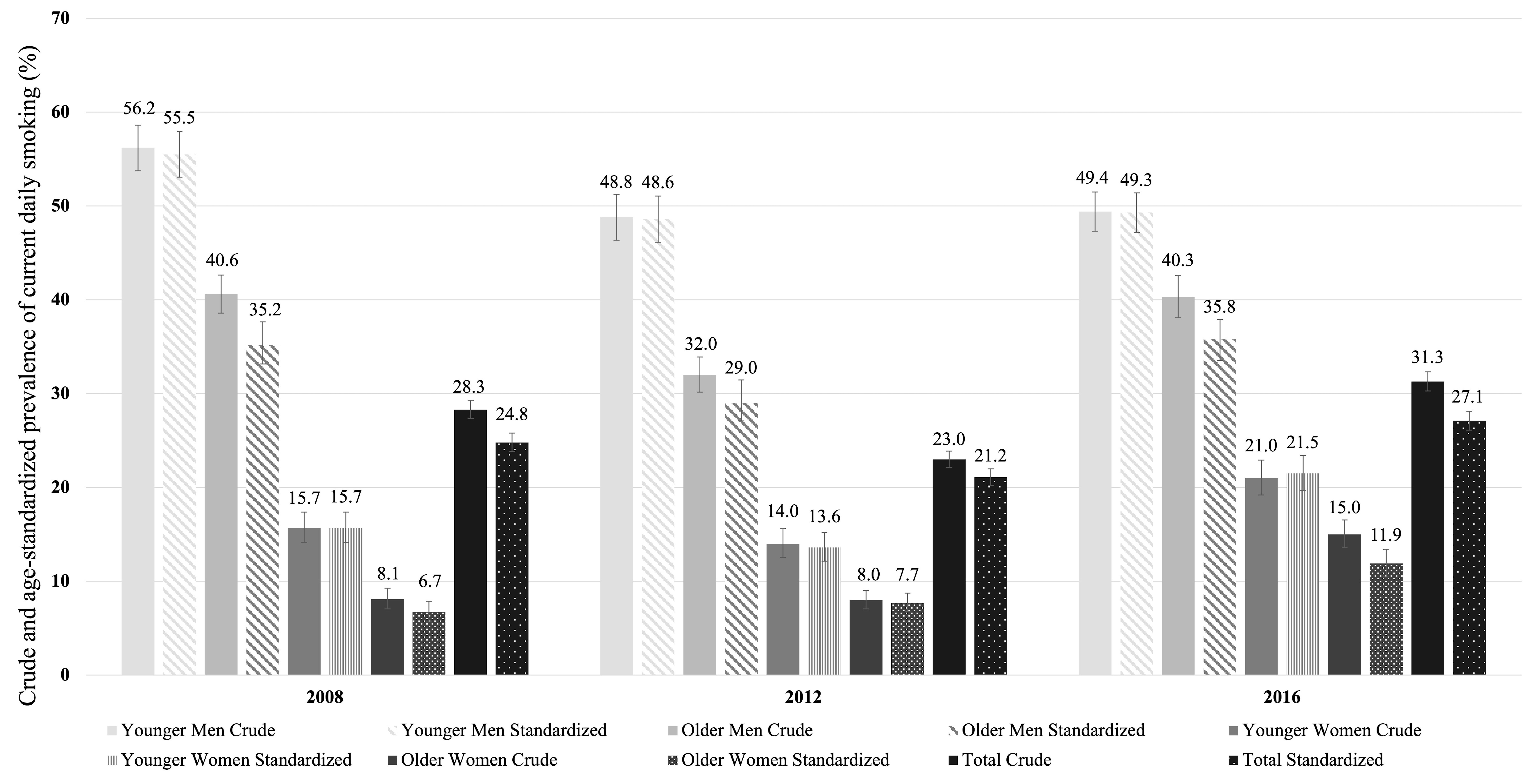

Supplement: Supplementary file 1 — Additional file 1: Supplementary Figure 1 Crude and age-standardized prevalence with 95%CIs of current daily smoking status by sex, age group and year. [file 12889_2021_12200_MOESM1_ESM.jpg]
