# Supplementary material for: Trends in socioeconomic inequalities in smoking in Turkey from 2008 to 2016
Source: BMC Public Health. 2021 Nov 20;21:2128. doi: 10.1186/s12889-021-12200-x (PMC8605534; doi:10.1186/s12889-021-12200-x)
Supplement: Supplementary file 2 — Additional file 2: Supplementary Table 1 The details about the data excluded from the study for each year. [file 12889_2021_12200_MOESM2_ESM.docx]

**Supplementary Table 1** The details about the data excluded from the study for each year

|  | **2008** | **2012** | **2016** |
| --- | --- | --- | --- |
| **Completed individual interviews** | 9030 | 9851 | 8760 |
| **Respondents below 20 years of age** | 541 | 651 | 392 |
| **Occasional smokers** | 299 | 280 | 170 |
| **Missing data** | 12 | 5 | 83 |
| **Remaining data** | 8178 | 8915 | 8115 |
