# Supplementary material for: Trends in socioeconomic inequalities in smoking in Turkey from 2008 to 2016
Source: BMC Public Health. 2021 Nov 20;21:2128. doi: 10.1186/s12889-021-12200-x (PMC8605534; doi:10.1186/s12889-021-12200-x)
Supplement: Supplementary file 3 — Additional file 3: Supplementary Table 2 Odds ratios for current daily smoking for women according to socioeconomic indicators by age group and survey year. [file 12889_2021_12200_MOESM3_ESM.docx]

**Supplementary Table 2** Odds ratios for current daily smoking for women according to socioeconomic indicators by age group and survey year

|  | **Younger Women (20-39 years)** | | | **Older Women (40 and above)** | | |
| --- | --- | --- | --- | --- | --- | --- |
|  | **2008** | **2012** | **2016** | **2008** | **2012** | **2016** |
|  | OR (95% CI) | OR (95% CI) | OR (95% CI) | OR (95% CI) | OR (95% CI) | OR (95% CI) |
| **Wealth** |  |  |  |  |  |  |
| Highest | **2.39 (1.49-3.86)** | **4.01 (1.82-8.84)** | 1.01 (0.46-2.23) | **1.76 (1.01-3.04)** | 1.60 (0.84-3.03) | **2.95 (1.03-8.47)** |
| Second highest | **2.29 (1.46-3.59)** | **3.85 (1.82-8.18)** | 0.97 (0.45-2.06) | **1.84 (1.11-3.06)** | **2.28 (1.27-4.08)** | 2.70 (0.96-7.63) |
| Middle | **2.14 (1.36-3.36)** | **3.44 (1.63-7.27)** | 1.38 (0.65-2.93) | 0.98 (0.56-1.71) | **2.17 (1.22-3.87)** | 2.63 (0.93-7.44) |
| Second lowest | 1.14 (0.69-1.90) | **2.67 (1.25-5.74)** | 1.12 (0.52-2.41) | 0.89 (0.47-1.69) | 1.57 (0.85-2.89) | **3.15 (1.09-9.07)** |
| Lowest (ref) | 1.00 | 1.00 | 1.00 | 1.00 | 1.00 | 1.00 |
| **Education** |  |  |  |  |  |  |
| High education | **3.41 (2.07-5.63)** | **2.35 (1.39-3.98)** | 1.88 (0.87-4.04) | **7.43 (4.55-12.15)** | **4.67 (2.89-7.55)** | **3.23 (2.00-5.21)** |
| Moderate education | **1.80 (1.14-3.01)** | 1.48 (0.88-2.49) | 1.15 (0.53-2.49) | **2.39 (1.56-3.66)** | **2.68 (1.77-4.05)** | 1.43 (0.91-2.24) |
| Low education (ref) | 1.00 | 1.00 | 1.00 | 1.00 | 1.00 | 1.00 |
| **Occupation** |  |  |  |  |  |  |
| Unemployed | 2.34 (0.90-6.07) | 1.98 (0.85-4.62) | 1.53 (0.78-2.99) | **2.77 (1.70-4.49)** | 1.76 (0.37-8.53) | 1.05 (0.22-4.89) |
| Nongovernment employee | **2.02 (1.49-2.73)** | **2.31 (1.68-3.17)** | **2.10 (1.57-2.80)** | **4.06 (2.54-6.48)** | **2.45 (1.51-3.95)** | **3.73 (2.57-5.40)** |
| Self-Employed | 1.68 (0.82-3.43) | 1.01 (0.51-2.02) | 0.94 (0.36-2.50) | 1.76 (0.77-3.99) | 0.78 (0.40-1.51) | **3.22 (1.58-6.55)** |
| Government employee | - | 1.31 (0.77-2.22) | 1.51 (0.96-2.36) | - | **2.37 (1.25-4.50)** | **2.54 (1.34-4.80)** |
| Homemaker/retired/student (ref) | 1.00 | 1.00 | 1.00 | 1.00 | 1.00 | 1.00 |
| **Place of residence** |  |  |  |  |  |  |
| Urban | **3.07 (2.31-4.06)** | **2.30 (1.72-3.08)** | **3.18 (1.74-5.80)** | **2.87 (2.06-4.00)** | **3.96 (2.89-5.41)** | **2.93 (1.76-4.88)** |
| Rural (ref) | 1.00 | 1.00 | 1.00 | 1.00 | 1.00 | 1.00 |
