# Supplementary material for: Trends in socioeconomic inequalities in smoking in Turkey from 2008 to 2016
Source: BMC Public Health. 2021 Nov 20;21:2128. doi: 10.1186/s12889-021-12200-x (PMC8605534; doi:10.1186/s12889-021-12200-x)
Supplement: Supplementary file 4 — Additional file 4: Supplementary Table 3 Odds ratios for current daily smoking for men according to socioeconomic indicators by age group and survey year. [file 12889_2021_12200_MOESM4_ESM.docx]

**Supplementary Table 3** Odds ratios for current daily smoking for men according to socioeconomic indicators by age group and survey year

|  | **Younger Men (20-39 years)** | | | **Older Men (40 and above)** | | |
| --- | --- | --- | --- | --- | --- | --- |
|  | **2008** | **2012** | **2016** | **2008** | **2012** | **2016** |
|  | OR (95% CI) | OR (95% CI) | OR (95% CI) | OR (95% CI) | OR (95% CI) | OR (95% CI) |
| **Wealth** |  |  |  |  |  |  |
| Highest | **0.59 (0.41-0.86)** | 1.18 (0.77-1.81) | 0.85 (0.49-1.48) | **0.63 (0.47-0.85)** | **0.58 (0.41-0.83)** | 0.76 (0.39-1.50) |
| Second highest | 0.87 (0.62-1.22) | 0.95 (0.65-1.40) | 1.02 (0.60-1.72) | 0.78 (0.59-1.04) | **0.63 (0.46-0.87)** | 0.99 (0.52-1.90) |
| Middle | 0.92 (0.65-1.29) | 1.16 (0.80-1.68) | 1.30 (0.77-2.20) | 0.86 (0.65-1.14) | **0.72 (0.52-0.99)** | 0.94 (0.49-1.80 |
| Second lowest | 1.01 (0.70-1.45) | 1.06 (0.73-1.56) | 1.10 (0.65-1.89) | 1.03 (0.76-1.41) | 0.81 (0.58-1.14) | 1.11 (0.57-2.15) |
| Lowest (ref) | 1.00 | 1.00 | 1.00 | 1.00 | 1.00 | 1.00 |
| **Education** |  |  |  |  |  |  |
| High education | **0.49 (0.26-0.91)** | 0.71 (0.37-1.38) | 0.66 (0.15-2.95) | **0.64 (0.45-0.91)** | 1.06 (0.93-1.26) | 0.75 (0.43-1.32) |
| Moderate education | 0.73 (0.40-1.36) | 1.16 (0.60-2.26) | 0.85 (0.19-3.85) | 0.84 (0.63-1.13) | 1.18 (1.12-2.27) | 0.95 (0.56-1.61) |
| Low education (ref) | 1.00 | 1.00 | 1.00 | 1.00 | 1.00 | 1.00 |
| **Occupation** |  |  |  |  |  |  |
| Unemployed | 1.30 (0.75-2.25) | **2.68 (1.59-4.54)** | 1.21 (0.82-1.77) | 0.95 (0.58-1.57) | **2.66 (1.52-4.65)** | **2.25 (1.40-3.61)** |
| Nongovernment employee | 1.40 (0.88-2.22) | **2.27 (1.48-3.46)** | **1.36 (1.03-1.80)** | 0.82 (0.48-1.40) | **1.45 (1.09-1.92)** | **1.66 (1.22-2.26)** |
| Self-Employed | 1.61 (0.99-2.64) | **1.73 (1.09-2.76)** | **2.11 (1.48-3.02)** | 1.21 (0.73-2.01) | **1.64 (1.27-2.11)** | **1.90 (1.35-2.68)** |
| Government employee | - | 1.18 (0.72-1.93) | 1.01 (0.69-1.47) | - | 0.84 (0.59-1.21) | 1.32 (0.87-1.99) |
| Homemaker/retired/student (ref) | 1.00 | 1.00 | 1.00 | 1.00 | 1.00 | 1.00 |
| **Place of residence** |  |  |  |  |  |  |
| Urban | 1.21 (0.98-1.51) | 1.15 (0.94-1.40) | 0.88 (0.65-1.18) | 1.14 (0.95-1.36) | 0.90 (0.75-1.08) | 0.93 (0.71-1.22) |
| Rural (ref) | 1.00 | 1.00 | 1.00 | 1.00 | 1.00 | 1.00 |
